# Supplementary material for: Co-Expression of Androgen Receptor and Cathepsin D Defines a Triple-Negative Breast Cancer Subgroup with Poorer Overall Survival
Source: Cancers (Basel). 2020 May 15;12(5):1244. doi: 10.3390/cancers12051244 (PMC7281089; doi:10.3390/cancers12051244)
Supplement: Supplementary file 1 [file cancers-12-01244-s001.pdf]

cancers

Co-Expression of Androgen Receptor and Cathepsin D Defines a Triple-Negative Breast Cancer Subgroup with Poorer Overall Survival

Supplementary

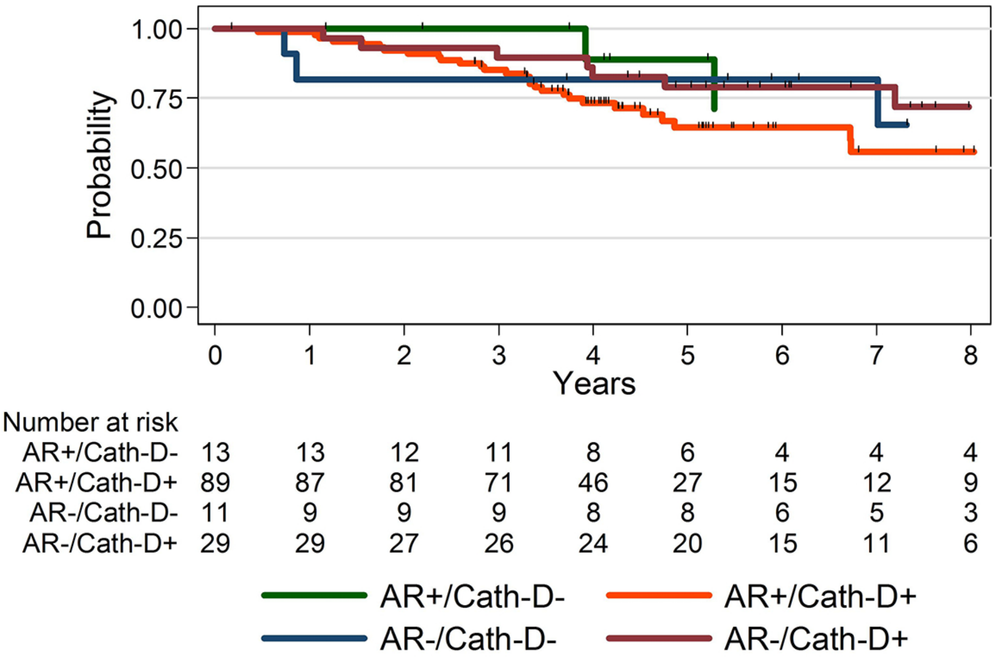

Figure S1. Overall survival in patients with non-metastatic TNBC (n=142) according to the AR and Cath-D expression profiles (four sub-groups).

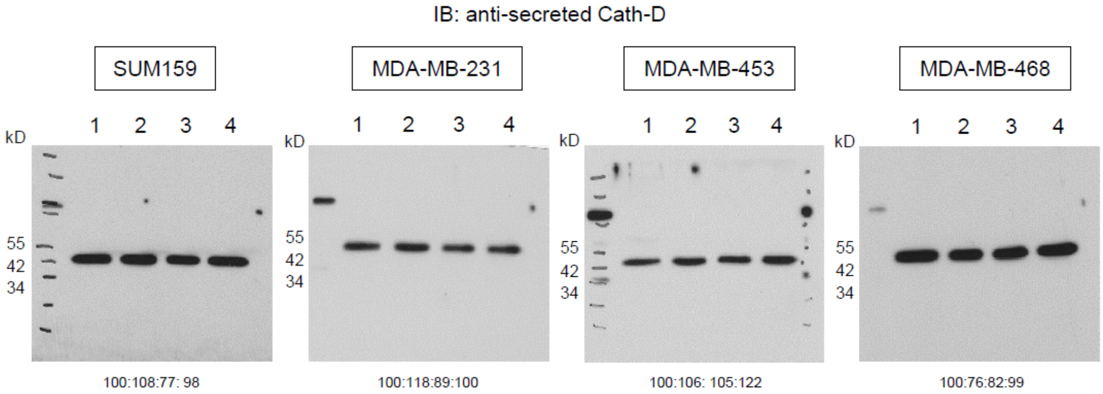

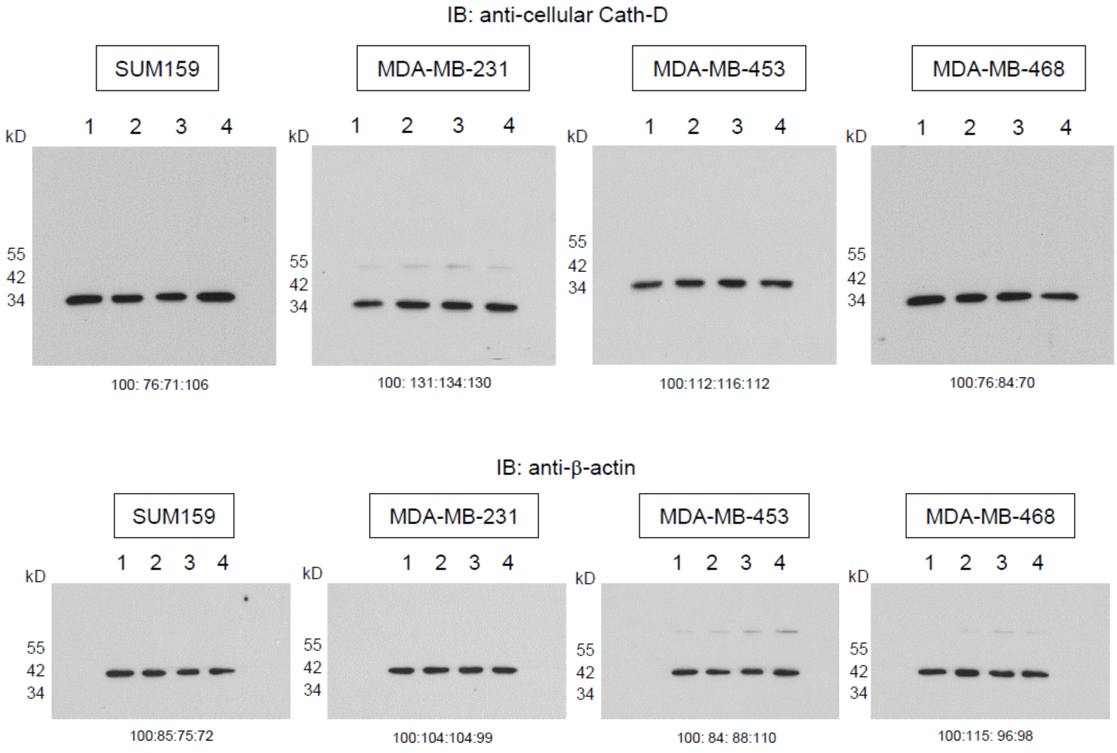

Figure S2. The whole western blot images of Figure.
